# Post-Disturbance Genetic Changes: The Impact of the 2010 Mega-Earthquake and Tsunami on Chilean Sandy Beach Fauna

**DOI:** 10.1038/s41598-019-50525-1

**Published:** 2019-10-02

**Authors:** Antonio Brante, Garen Guzmán-Rendón, Erwin M. Barría, Marie-Laure Guillemin, Iván Vera-Escalona, Cristián E. Hernández

**Affiliations:** 1 Departamento de Ecología, Facultad de Ciencias, Universidad Católica de la Santísima Concepción, Concepción, Chile; 2 Centro de Investigación en Biodiversidad y Ambientes Sustentables (CIBAS), Universidad Católica de la Santísima Concepción, Concepción, Chile; 3 Programa de Doctorado en Sistemática y Biodiversidad, Departamento de Zoología, Facultad de Ciencias Naturales y Oceanográficas, Universidad de Concepción, Concepción, Chile; 4 Laboratorio de Ecología Evolutiva y Filoinformática, Departamento de Zoología, Facultad de Ciencias Naturales y Oceanográficas, Universidad de Concepción, Concepción, Chile; 5 Instituto de Ciencias Ambientales y Evolutivas, Facultad de Ciencias, Universidad Austral de Chile, Casilla 567, Valdivia, Chile; 6 CNRS, Sorbonne Universités, UPMC University Paris VI, UMI 3614, Evolutionary Biology and Ecology of Algae, Station Biologique de Roscoff, CS 90074, Place G. Tessier, 296888 Roscoff, France; 7 Departamento de Ciencias Básicas, Universidad Santo Tomás, Osorno, Chile

**Keywords:** Molecular ecology, Marine biology

## Abstract

Earthquake/tsunamis can have profound impacts on species and their genetic patterns. It is expected that the magnitude of this impact might depend on the species and the time since the disturbance occurs, nevertheless these assumptions remain mostly unexplored. Here we studied the genetic responses of the crustacean species *Emerita analoga*, *Excirolana hirsuticauda*, and *Orchestoidea tuberculata* to the 27F mega-earthquake/tsunami that occurred in Chile in February 2010. mtDNA sequence analyses revealed a lower haplotype diversity for *E. analoga* and *E. hirsuticauda* in impacted areas one month after the 27F, and the opposite for O. *tuberculata*. Three years after the 27F we observed a recovery in the genetic diversity of *E. analoga* and *E. hirsuticauda* and decrease in the genetic diversity in *O. tuberculata* in 2/3 of sampled areas. *Emerita analoga* displayed decrease of genetic differentiation and increase in gene flow explained by long-range population expansion. The other two species revealed slight increase in the number of genetic groups, little change in gene flow and no signal of population expansion associated to adult survival, rapid colonization, and capacity to burrow in the sand. Our results reveal that species response to a same disturbance event could be extremely diverse and depending on life-history traits and the magnitude of the effect.

## Introduction

Large-scale natural disturbances can have profound impacts on ecosystems, affecting several ecological and fitness traits of key species through the reduction in population sizes and habitat destruction (e.g.^1–4^). For example, mega natural disturbances, such as tsunamis, earthquakes, fires, and hurricanes can drive long-term variations in the abundance and distribution of species, community dynamics and ecosystem processes in terrestrial and marine ecosystems^1–14^. While the extension of these disturbances is short, they can have deep effects on species. Nevertheless, extreme disturbances not only affect species on an ecological level, but also a genetic level. Provided that genetic diversity is linked to the evolutionary potential of a species to respond to a changing environment, the genetic composition of a population plays an important role for its persistence, since populations with more genetic diversity are less prone to extinction^15–17^. Thus, evaluating the impact of disturbances on species’ genetic diversity and their responses on short and long temporal scales is fundamental to understanding the factors and mechanisms that determine population and ecosystem resilience.

Empirical evidence evaluating populations’ genetic responses to disturbances are scarce and mostly focused on the effects of rapid anthropogenic destruction of forest ecosystems (see^18^). The existing evidence has shown that impacts on genetic diversity appear to be idiosyncratic, depending not only on the magnitude of the disturbance, but also on life history traits associated with species’ breeding systems and their dispersal capacities^18^. For example, genetic studies have demonstrated a loss in genetic diversity in the tropical tree *Pithecellobium elegans* after the destruction of its habitat^19^. On the contrary, no effects were observed in any of the genetic diversity indices of the tree *Swietenia macrophylla* as a consequence of a century of logging^20^. This patterns in *S. macrophylla* was explained by the increase in pollen flow and range expansion as a result of logging, which reduced the loss of diversity due to this species’ dispersal capacity. In one of the few studies available on marine species, authors reported that the genetic diversity of populations of the kelp *Lessonia nigrescens* in northern Chile was reduced by up to 50% after the El Niño–Southern Oscillation event of 1982/83^21^. Since then, this species has shown a slow genetic recovery that could be explained by its low dispersal potential. On the other hand, authors studying the intertidal goby *Chaenogobius annularisthe* and the mud snail *Batillaria attramentaria* found no evidence of loss in genetic diversity as a consequence of the 2011 Tohoku earthquake and tsunami^22,23^. Provided that the scale, magnitude and duration of each disturbance varies widely depending upon the study, generalisations about species’ responses to mega perturbations are difficult to draw. To overcome this limitation, it has been proposed that, when possible, studies should examine the genetic responses of multiple species with contrasting ecological and life history traits to the same ecological disturbance^18^.

On February 27^th^, 2010 (27F), the southern-central coast of Chile was struck by a mega-earthquake (8.8 M_w_) and tsunami. The earthquake’s rupture zone covered mainly a region localized between 35° and 37°S^24^, generating changes in the elevation of the coastline of up to 3 m. As a consequence of the earthquake, massive mortality of intertidal flora and fauna occurred in localities with extreme coastal uplift^3,4^. In addition, the tsunami produced by the earthquake significantly affected nearshore marine life, devastating hundreds of kilometres of coast^3,4,12^. The magnitude of the tsunami was variable along the Chilean coast, according to the distance from the epicentre and orientation of the coastline^25^. In a study of the ecological consequences of the 27F earthquake authors found that the mortality of intertidal invertebrates on sandy beaches was positively correlated with tsunami height^4^. This study suggested that even in the absence of changes in coastal height, the ecological effects of the 27F tsunami on Chilean marine ecosystems could be significant; similar observations have been reported for subtidal macrofauna in other coastal areas of the world^26–29^.

**Figure 1:**
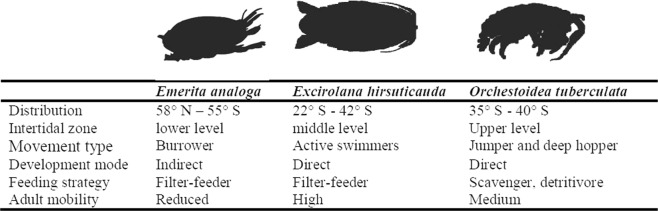
Main traits and modalities of *Emerita analoga*, *Excirolana hirsuticauda* and *Orchestoidea tuberculata*.

Here we evaluated the population genetic response to the 27F mega-earthquake and tsunami in three common crustacean species of Chilean sandy beach ecosystems: *Emerita analoga*, *Excirolana hirsuticauda*, and *Orchestoidea tuberculata*. These species have diverse life history traits, which have differential consequences on their dispersal ability (Fig. 1). The sand mole *E. analoga* is a widely distributed decapod crustacean (58°N to 55°S) inhabiting the lower intertidal zone with a meroplanktonic life cycle and high dispersal ability whose larvae can be found in Antarctica and Polynesia, although adults are only found along the coasts of Chile and Peru^30,31^. The cirolanid isopod *E. hirsuticauda* is a conspicuous endemic component of the middle shore intertidal level of Chilean sandy beaches between 22°S and 42°S^32,33^ with juveniles and adult individuals being active swimmers^34,35^. The talitrid amphipod *O. tuberculata* inhabits the upper level of the intertidal zone^36–40^ of Chile (35°S–40°S), with females incubating their offspring to the juvenile stage and low dispersal potential. These three species were strongly affected by the 27F mega-earthquake, but their responses varied intraspecific and interspecifically depending upon the particular impacts experienced by the diverse populations (variation of uplift level and tsunami height depending on the beach) and the species studied^4,41^. For instance, ecological studies have shown that *E. analoga* was highly affected during the 27F, but presented a low colonization following the 27F; a contrasting pattern was observed in *E. hirsuticauda* and *O. tuberculata*, where a rapid post-disturbance colonization was recorded^4,41,42^.

**Figure 2:**
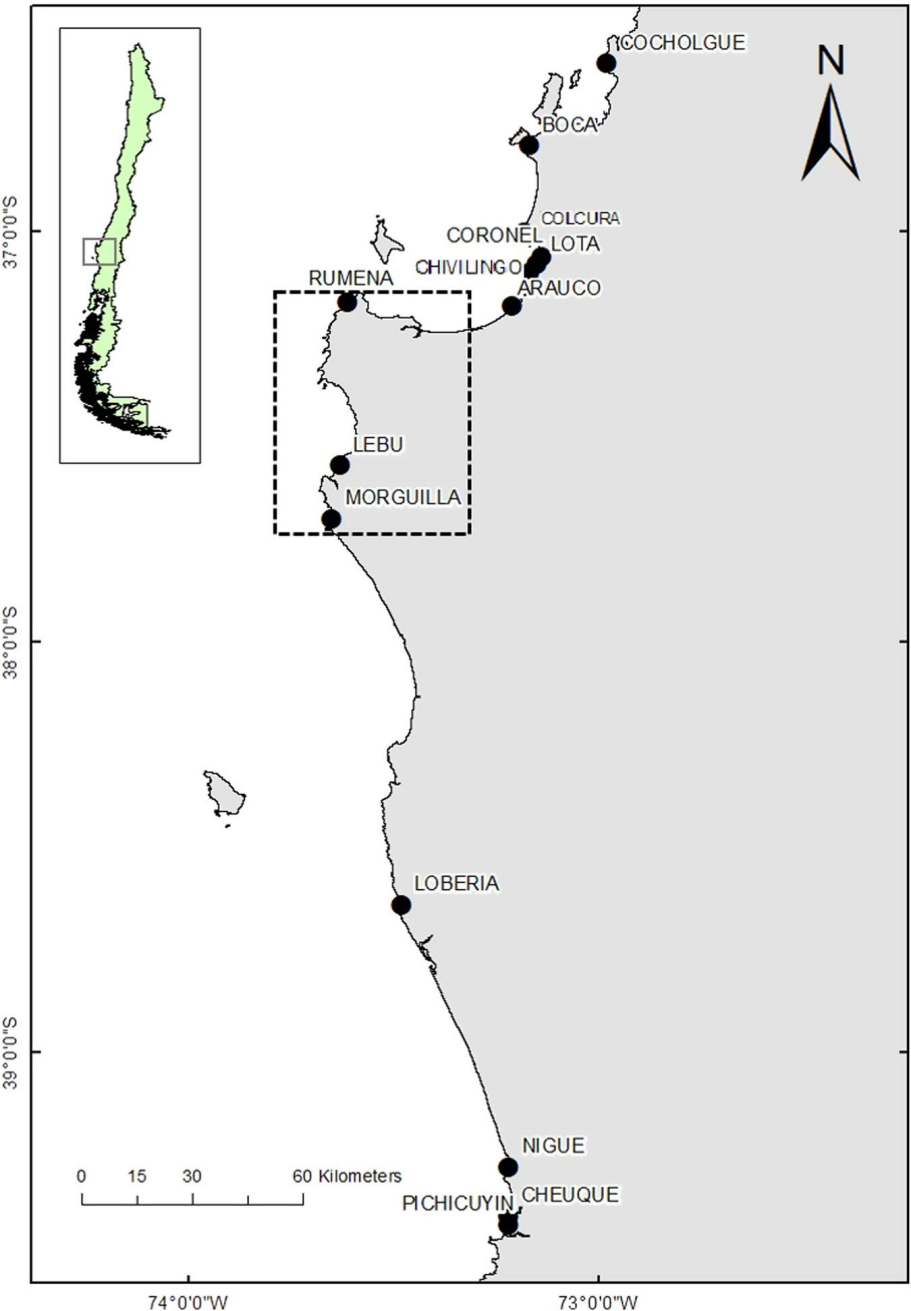
Map of sampling sites along the southern-central Chilean coast (36–39°S). The area affected by the tsunami and earthquake is shown in the dashed-lined box.

In this study, we assessed the effects of the 27F mega disturbance on genetic diversity and population structure of *E. analoga*, *E. hirsuticauda*, and *O. tuberculata* along the Chilean coast. Comparisons among sampled localities were made through time and among highly impacted areas (37°09′–37°42′S, Fig. 2) and non-impacted control areas located south and north of the disturbed area. We hypothesised that higher impacts on population genetic traits would be observed in *E. analoga* given its lower adult mobility in comparison to the other two species. However, a fast recovery of population genetic diversity was also expected in this species given the presence of a free larval stage with a high dispersion potential.

## Results

The sequence alignment length and concatenation of both genes was 875 bp for T1 and T2, respectively, in *E. analoga*, 1070 bp (T1 and T2) in *E. hirsuticauda*, and 1078 bp (T1 and T2) in *O. tuberculata* (Genbank Accession number MK914652 - MK917441). No evidence of stop codons were detected in the COI gene, no substitution saturation was found for any species or sampling time, indicating that data sets were suitable for all of the subsequent genetic analyses.

### Genetic structure

**Figure 3:**
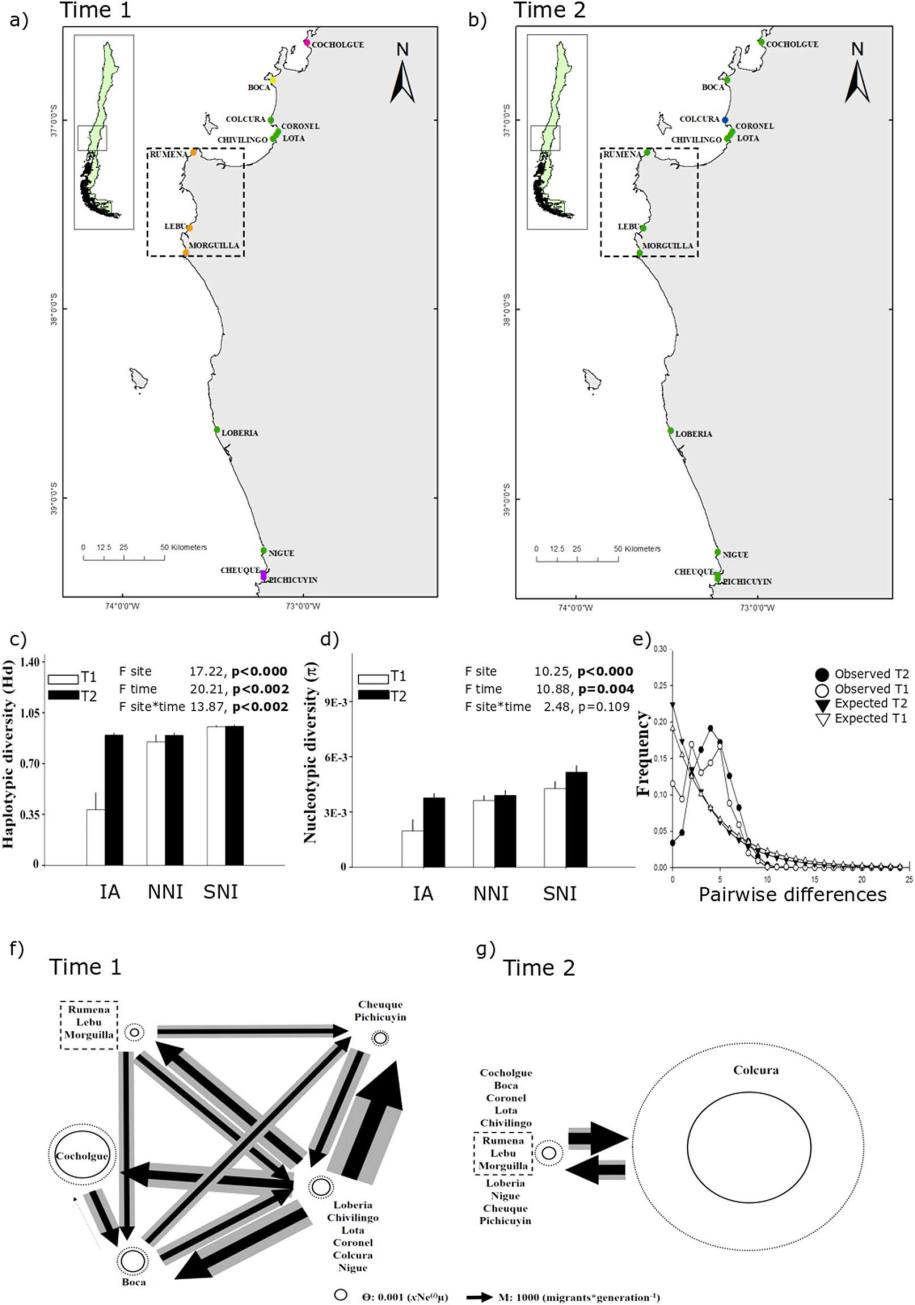
Genetic clusters identified with GENELAND (each color represents a genetic cluster) at Time 1 (T1; **a**) and Time 2 (T2; **b**), mean haplotype diversity (**c**) and nucleotide diversity + SD (**d**) including genetic differences and results of AMOVA analyses among sites, times (T1 and T2), and sites-times, mismatch distribution (**e**), and gene flow estimated with MIGRATE-n for *E. analoga* at T1 (**f**) and T2 (**g**). Arrows’ width represents the amount of immigrant numbers, circles’ size represents the value of Ne. NNI: northern non-impacted area, IA: impacted area and SNI: southern non-impacted area.

**Figure 4:**
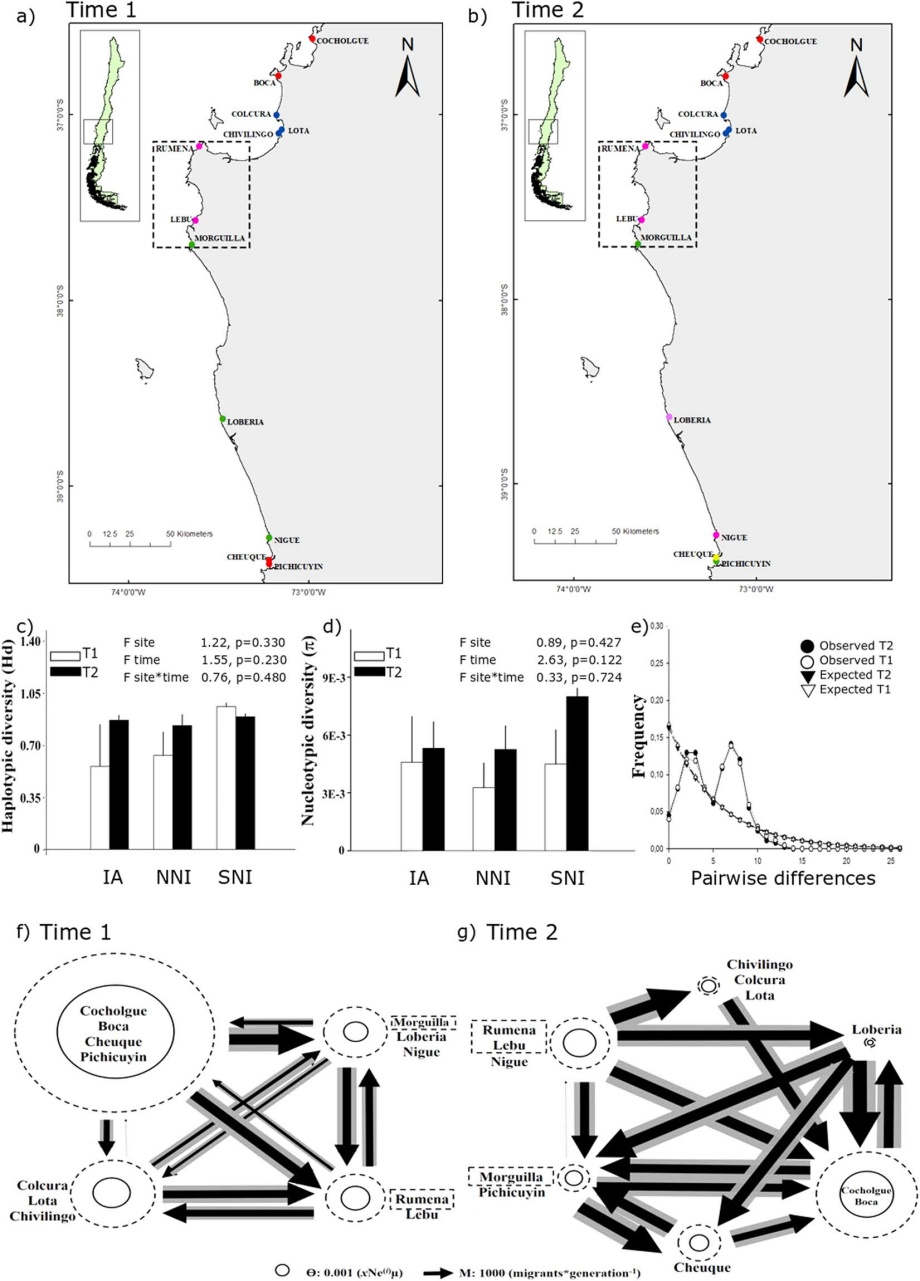
Genetic clusters identified with GENELAND (each color represents a genetic cluster) at Time 1 (T1; **a**) and Time 2 (T2; **b**), mean haplotype diversity (**c**) and nucleotide diversity + SD (**d**) including genetic differences and results of AMOVA analyses among sites, times (T1 and T2), and sites-times, mismatch distribution (**e**), and gene flow estimated with MIGRATE-n for *E. hirsuticauda* at T1 (**f**) and T2 (**g**). Arrows’ width represents the amount of immigrant numbers, circles’ size represents the value of Ne. NNI: northern non-impacted area, IA: impacted area and SNI: southern non-impacted area.

**Figure 5:**
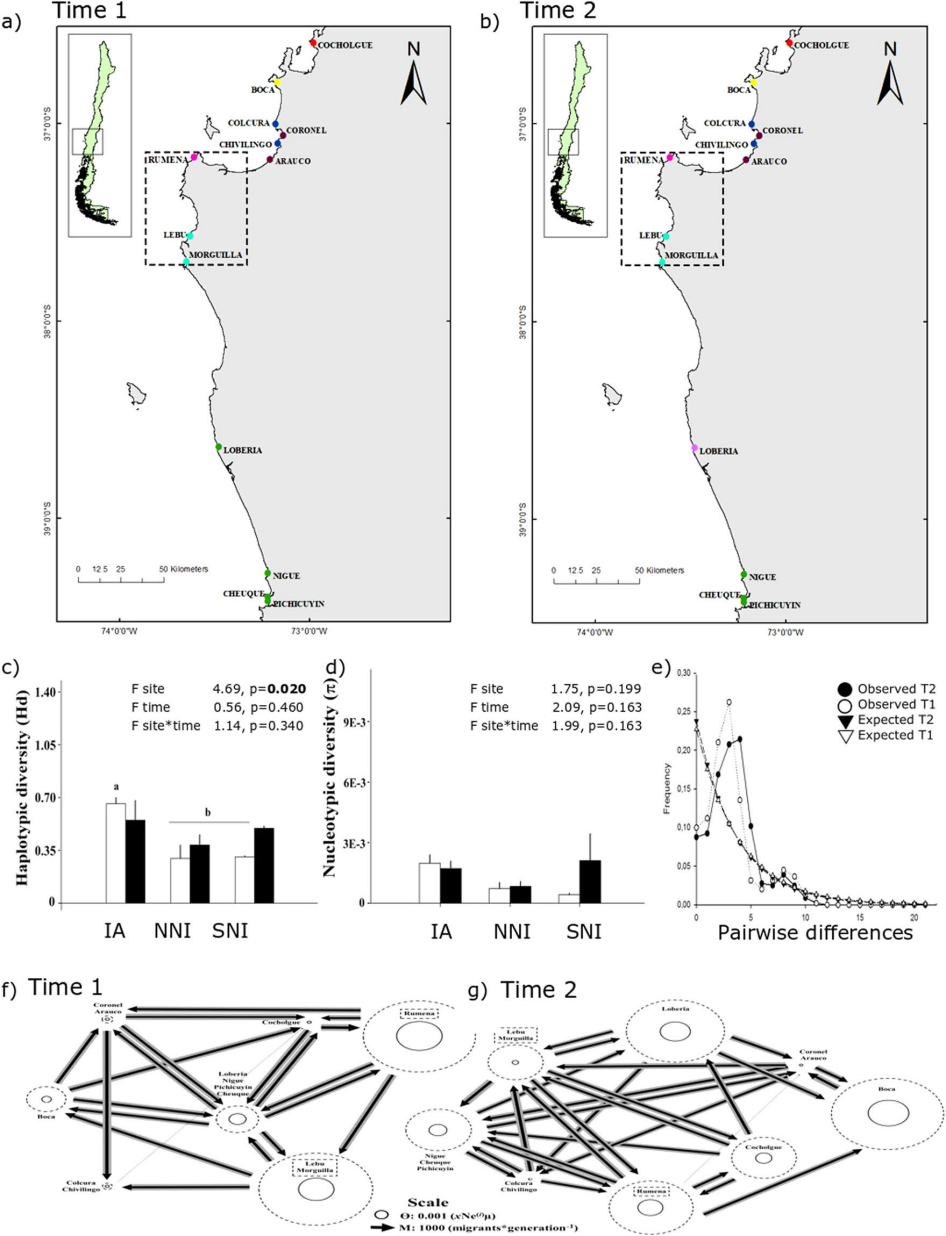
Genetic clusters identified with GENELAND (each color represents a genetic cluster) at Time 1 (T1; **a**) and Time 2 (T2; **b**), mean haplotype diversity (**c**) and nucleotide diversity + SD (**d**) including genetic differences and results of AMOVA analyses among sites, times (T1 and T2), and sites-times, mismatch distribution (**e**), and gene flow estimated with MIGRATE-n for *O. tuberculata* at T1 (**f**) and T2 (**g**). Arrows’ width represents the amount of immigrant numbers, circles’ size represents the value of Ne. NNI: northern non-impacted area, IA: impacted area and SNI: southern non-impacted area.

The Bayesian approach of spatial clustering based on Geneland analyses showed changes in the genetic structure of all of the studied species through time (Figs 3–5). The number of genetic clusters decreased throughout time in *E. analoga* (from K = 5 to K = 2) and slightly increased in *E. hirsuticauda* (from K = 4 to K = 6) and *O. tuberculata* (from K = 7 to K = 8; Figs 3a,b–5a,b). Probabilities for each genetic cluster were greater than 0.5 in most cases, except for *E. hirsuticauda* in T2, where the probabilities ranged between 0.2–0.4. *Emerita analoga* populations in the impacted area were well differentiated in T1, while they were part of the largest cluster in T2 (Fig. 3a,b). *Excirolana hirsuticauda* and *O. tuberculata* in the impacted area maintained the same genetic differentiation in southern and northern non-impacted areas between T1 and T2 (Figs 4a,b and 5a,b). The AMOVA comparing the zones with different levels of impact showed that *E. analoga* and *E. hirsuticauda* have different spatiotemporal patterns of haplotype structure than *O. tuberculata*. In both periods of analysis, the genetic-population structure of the first two species was mainly explained by the haplotype intrapopulation variation, followed by the haplotype variations between populations within each zone. In contrast, for *O. tuberculata*, the greatest contribution to the haplotype structure in both periods was explained by the variation between populations within zones, followed by intrapopulation variation; although this last level did not constitute a significant determinant of haplotype structuring (Table S1).

### Genetic variation

Mean haplotype diversity (Hd) in *E. analoga* populations from the impacted area was lower than Hd from northern and southern non-impacted areas at T1 (Table S2). At T2, Hd from impacted areas became very similar to both non-impacted areas at T2. Hd in *E. analoga* was significantly different between sites (p < 0.001), time (p < 0.002), and site*time (p < 0.002; Fig. 3c). For *E. hirsuticauda*, no significant differences were found in the mean value of Hd for either of the two factors (site and sampling time) nor for their interaction site*time (p < 0.330, p < 0.230, p < 0.480; Fig. 4c; Table S2). Mean Hd in *O. tuberculata* in the impacted area was higher than Hd in northern and southern non-impacted areas at T1 (p < 0.02; Fig. 5c; Table S2). At T2, the mean Hd in the impacted area was still higher than in both northern and southern non-impacted areas, but northern non-impacted areas showed less Hd than southern non-impacted areas. The lowest mean value of nucleotide diversity (π) for *E. analoga* was observed in the impacted area at T1, and increased significantly between T1 and T2 (Fig. 3d). At T2, π of this species in impacted and northern non-impacted areas was similar, while it was higher in southern non-impacted areas. Nucleotide diversity (π) in *E. analoga* showed significant differences for both the factor area and sampling time (p < 0.001 and p = 0.004 respectively), but not for their interaction (Fig. 3d). For *E. hirsuticauda*, π did not differ for either of the two factors or for the interaction between them (Fig. 4d). Nucleotide diversity of *O. tuberculata* was lower than in the other two species (Fig. 4d). At T1, π was higher among populations in impacted than in non-impacted areas. At T2, a decrease in π in populations in impacted were observed while values of π were maintained in northern non-impacted areas. Contrastingly, an increase in π among southern non-impacted areas between T1 and T2 was observed. In *O. tuberculata*, differences in π values were non-significant among sites, times and sites*times (Fig. 5d). Differences between pairs of sequences estimated using the mismatch analyses showed a unimodal distribution for *E. analoga* at T2, a signal of population expansion (Fig. 3e). Contrastingly, a bimodal distribution for *E. hirsuticauda* and *O. tuberculata* in T1 as well as in T2 was observed (Fig. 4e,e).

### Gene flow analyses

In all cases, the migration analysis supported the panmictic model (BF_(Panmictic-Full Model)_ >5.0) and showed temporal changes in effective population size (Ɵ) and migration rate among clusters (M). In *E. analoga* the cluster composed of populations in the impacted area had the lowest value of effective population size in T1 (Ɵ: 0.43E-3 ± 0.37E-3; Fig. 3f). This magnitude was approximately 8 and 6 times lower than the maximum values of Ɵ at T1, which corresponded to the Cocholgüe (Ɵ: 3.42E-3 ± 3.39E-3) and Boca (Ɵ: 3.42E-3 ± 3.39E-3) clusters, both located in the northern non-impacted area. At T2, the effective population size of the cluster formed by the population of Colcura (Ɵ: 9.49E-3 ± 3.28E-3) was approximately 9.1 times greater than the Ɵ value of the cluster combining the remaining populations (Ɵ: 1.04E-3 ± 1.09E-3; Fig. 3g). Gene flow was moderate to high among all populations at T1, with the highest number of immigrants occurring in non-impacted areas in the southernmost localities. In T2, gene flow was high and mostly symmetric for the two identified clusters. Despite the difference in Ɵ, the migration rate from the largest cluster to Colcura (M: 3901 ± 1539 migrants*generation^−1^) was 12% greater than the gene flow from Colcura to the largest cluster (M: 2731 ± 1421 migrants*generation^−1^; Fig. 4g).

In *E. hirsuticauda*, the two clusters that contained the populations of the impacted area, the Lebu-Rumena and Lobería-Nigue-Morhuilla clusters, presented the lowest values of effective population size in T1 (Ɵ: 2.94E-3 ± 3.27E-3 and Ɵ: 2.54E-3 ± 4.08E-3 respectively; Fig. 4f). These Ɵvalues were about 75% lower than the effective population size of the cluster formed by the extreme populations from both the northern and southern non-impacted areas (Cocholgüe-Boca-Cheuque-Pichicuyín cluster), and approximately 24% lower than Ɵ of the Chivilingo-Colcura-Lota cluster. At T2, the two northernmost locations (Cocholgüe and Boca) constituted the cluster with the largest population size (Ɵ = 4.92E-3 ± 5.35E-3), followed by three clusters combining localities from the affected area and the southern non-affected area. Of these three clusters, the individuals of the affected populations in Lebu and Rumena were genetically grouped with Nigue, forming the cluster with the second largest population size Ɵ: 2.96E-3 ± 2.50E. At T1, gene flow among all populations was moderate to low and mostly symmetric (Fig. 4f), while it was moderate and symmetric at T2 (Fig. 4g).

In *O. tuberculata* at T1, individuals from localities belonging to the impacted area formed two clusters showing the highest values of population size (Rumena cluster Ɵ: 5.19E-3 ± 7.57E-3 and Lebu-Morgüilla cluster Ɵ: 4.00E-3 ± 8.91E-3; Fig. 5f). Values of Ɵ for these clusters were more than twice those estimated for the third largest cluster (Ɵ: 1.94E-3 ± 2.87E-3) composed of populations from the four localities of the southern non-impacted area. Of the eight genetic clusters observed at T2, two were located within the impacted area (Fig. 5g), with the Rumena cluster showing a larger population size (Ɵ: 2.97E-3 ± 7.02E-3) than the Lebu-Morgüilla cluster (Ɵ: 1.45E-3 ± 5.12E-3). However, Ɵ values of both of these clusters were smaller than those estimated in the Boca and Lobería clusters, which presented the largest population sizes (Ɵ: 4.83E-3 ± 8.55E-3 and Ɵ: 3.58E-3 ± 8.10E-3, respectively). Gene flow among all populations was symmetric and moderate to low at T1 (Fig. 5f) and moderate and symmetric at T2 (Fig. 5g).

## Discussion

Disturbance processes, such as earthquakes associated with coastal uplift and tsunamis, can shape the genetic patterns of coastal species (i.e. population structure, gene flow and diversity)^43^. When populations are suddenly impacted by a mega disturbance, their genetic diversity tends to decline and the rate of that decline depends mostly on the effective population size, the number of mutations occurring after the disruptive event, and the amount of new genetic variants in a population as a result of gene flow^44–46^. Nevertheless, not only the genetic attributes of populations can help to understand the genetic responses of species to a disruptive event; ecological traits also play an important role in explaining the direction and magnitude of changes observed in the genetic composition of species under perturbation. As a result of regional and local variations in habitat, magnitude of the disruptive event and ecological attributes characterizing affected communities, each population is expected to react uniquely to perturbations, revealing a unique history of bottleneck and recovery^18^. Our results, comparing the genetic consequences of the 27F earthquake/tsunami in three species with different history traits suggest that species’ responses to disruptive events can be extremely diverse, even when exposed to the same disturbance. These responses are highly depending on the combined effects of the main species traits and modalities such as movement type, mobility degree, and intertidal zone habitat occupied (Fig. 1).

The negative impacts of the combined effects of earthquake and tsunami on impacted areas through time is evident in the genetic parameters of *E. analoga*. The genetic diversity of *E. analoga* was lower among populations from impacted areas at T1, showing a significant increase by T2, reaching values similar to those found in non-impacted areas. After the earthquake, *E. analoga* showed a significant reduction in the number of populations; nonetheless, throughout time, as a consequence of gene flow and population expansion, *E. analoga* showed a higher genetic diversity at T2 when compared to T1. At T2 the largest cluster, including individuals from 13 of the 14 sampled localities, presented the lowest effective population size (*N*_*e*_) compared to the populations comprised of individuals from the single locality of Colcura. This lower *N*_*e*_ in the largest cluster could be explained by the observed population expansion signal and increase in the gene flow, leading to the homogenization of the 13 populations at T2, a pattern that has been well documented in theoretical and empirical studies^47^. Ecological studies analysing the effects of the 27F earthquake in *E. analoga* have suggested that this species was one of the most affected by the coastal uplift and tsunami during the 27 earthquake^4,41^. Consequently, the reduction of the genetic diversity of *E. analoga* through time could be the result of the combined effect of the removal of recruits and adults during the 27F tsunami as well as a source-sink dynamic. In addition, it has been noticed that the higher recruitment of *E. analoga* throughout the sampled area occurs near February^41^, thus the disturbance events from the 27F might have reduced the available habitat for new recruits. Provided that the massive adult mortality was a consequence of the coastal uplift, which may have wiped out most of the rare genetic variants in adults, a lower genetic diversity and higher population differentiation could be expected after the earthquake. However, our results suggest that three years after the disruptive event of the 27F, populations might have recovered, with new recruits settled, reducing the genetic differentiation, increasing the genetic diversity and revealing signals of population expansion in the mismatch analyses. This last finding could be the result of a recolonization of individuals from distant non-impacted areas, which can be explained by the high dispersal potential of *E. analoga* given its intermediate larval dispersal stage in areas like Polynesia and Antarctic waters^30,31^.

The genetic patterns observed in *E. hirsuticauda* are in line with the ones of other intertidal species^23^. This species showed no significant differences in either genetic diversity index at the two sampling times, in impacted and non-impacted areas. However, the analysis of population differentiation throughout time revealed a slight increase in population differentiation, no signal of population expansion, and an increase in gene flow along with a reduction of *N*_*e*_ in the southern non-impacted area at T2. A low or nil impact of mega disturbances has also been reported in other marine species. Though this is the first study assessing the effects of earthquakes/tsunamis on crustacean’s species, studies in other species can also be a matter of interest for contrasting results. For instance, along the coast of Japan, authors have noticed that the intertidal mud snail *Batillaria attramentaria* experienced a reduction in its effective population size (*N*_*e*_) after the 2011 Tohoko tsunami^23^. Nevertheless, the effect of this mass mortality produced no significant reduction in the allelic richness of this mud snail. These authors suggested that the lack of impact on this genetic diversity descriptor may have been the result of a combination of very large populations before the tsunami and a high recruitment of juveniles after the disturbance.

Although the general patterns revealed in our study found that at T2 *E. analoga* and *E. hirsuticauda* species decreased their Hd in impacted areas and increased their Hd in non-impacted areas, in *O. tuberculata* the highest Hd was recorded in the impacted area at T1. The same pattern was observed at T1 for π in *O. tuberculata*, but the increase in π in northern non-impacted areas was so high that it turned out to be the area with the highest Hd at T2. Similar to that observed in *E. hirsuticauda*, an increase in the number of populations was detected in *O. tuberculata* at T2, with the differentiated populations located within the southern non-impacted area. Although there are only a few of studies available linking ecology and genetics in sandy beach organisms exposed to disturbances, our results are on the line of previous results with talitrids^48–50^. Ecological studies have found a similar trend, with the species *E. hirsuticauda* and *O. tuberculata* showing similar abundances before and after the 27F earthquake^4^. Other authors have found that both species quickly recovered their abundance after the tsunami^51^, suggesting that the particular life history traits of these species may help to buffer the impacts of the mega-perturbation on their local populations. For instance, a high recovery of *Orchestoidea tuberculata* has been suggested to be related with its feeding strategy as scavengers and detritivorous (Fig. 1)^51^. For *E. hirsuticauda*, the lack of significant reductions in its density and genetic diversity could be explained by the high mobility of adults, and high dispersal potential of planktonic larvae. The swimming capacity of adults may allow them to resist the tsunami wave and then, relocate to a new site. In addition, the free larval stage has the capacity to rapidly colonize impacted areas as was suggested previously^42^. Contrary to E*. hirsuticauda*, *O. tuberculata* does not present an intermediate dispersal stage given that females incubate their offspring until they reach the juvenile stage (Fig. 1). However, adults have the capacity to jump and burrow up to 30 cm in the sand for shelter (Fig. 1)^34^, which together with their high resistance to desiccation could help this species to survive after disrupting events. Thus, it appear that *O. tuberculata* shows a high genetic and ecological resilience, possibly providing this species with advantageous adaptations to evade the direct effects of the tsunami and survive a coastal uplift and tsunami such as the 27F mega-earthquake.

Our study clearly reveal that species’ response to a major disturbance, in this case an earthquake and tsunami, depends on the ecology of each species; and that these distinct responses can influence the genetic diversity of species. The use of highly variable DNA sequences allowed to observe these changes in genetic diversity over a short period of time, only a few generations within each species. Whether DNA sequences can be used for recent processes has been the debate of multiple studies throughout the last decade (e.g.^52–54^), with no decisive answer. Here we have shown that recent massive disruptive events can actually be detected with DNA sequences, most likely due to the magnitude of the event. Nevertheless, it will be necessary to compare these results with other markers characterized by faster mutation rates, such as microsatellite or SNPs^53^, in order to confirm our conclusion. In a broader perspective, no hypotheses has been made yet on the ecologically meaningful units at which genetic patterns are revealed for sandy beach resident organisms. Our results suggest that we can detect changes in the genetic patterns of sandy beach organisms at population level after a disruptive event, even between relatively close populations. Although more observations are needed, this study can help future researchers to evaluate what major drivers explain the genetic diversity of species (with different life history traits) inhabiting areas frequently exposed to disruptive events.

Although areas suffering constant disturbances present important challenges for human populations, they also present interesting opportunities, since they act as natural laboratories to study changes in the ecological and genetic patterns of populations during recovery process after strong perturbations. It could be crucial to submit these areas to continuous sampling and monitoring in order to understand how species respond to changes in a landscape under recurrent perturbation regime. This information could then be used to predict what may happen to species facing natural or human-induced habitat modifications and thus provide better information for the conservation of species undergoing environmental disturbances.

## Methodology

### Individuals’ collection and laboratory protocols

Given that no samples were available before the mega-earthquake that occurred in February 2010, we used an impact-control approach. For this, we sampled 14 localities (sandy beach localities between 36°S and 39°S) with different levels of impacts, according to the uplift level and tsunami strength^4,14^. The magnitude of the impact was categorized in 3 areas: northern non-impacted area (NNI), impacted area (IA) and southern non-impacted area (SNI) (Fig. 2; Table S2). Samplings were carried out in March 2010 (T1), one month after the mega-earthquake, and repeated three years later in 2013 (T2), between February and June. All sampled localities were inside non-protected areas. Whole DNA was extracted from collected individuals using an already published protocol^55^ and its quality was evaluated in a ND 1000 spectrophotometer (NanoDrop Technologies). DNA templates were amplified for the mitochondrial genes COI and 16S using the PCR protocols described by other authors^56–58^. Amplified products were then sequenced in Macrogen Inc. (Seoul, Korea). Sequences of both genes were concatenated with Mesquite 3.4 software^59^ and translated to determine whether stop codons were present in the COI gene.

### Characterization of the sequence matrices

The potential existence of saturation^60^ and adjustment to a neutral model of mutation-drift equilibrium^61^ were assessed with the software DAMBE 6.4.100^62^ and DnaSP 6.0^63^, respectively. DnaSP was also used to calculate both haplotype and nucleotide diversity (Hd and π). For each species, mean Hd and π values were compared using a two-way Analysis of Variance (ANOVA), with two fixed factors: area with three levels (northern non-impacted area, impacted area and southern non-impacted area), and time with two levels (T1 and T2). When significant effects were detected, Tukey *a posteriori* tests were performed. All two-way ANOVAs were run in the STATISTICA 7.0 program^64^.

### Analysis of spatial genetic structure

GENELAND 4.08 was used to detect genetic discontinuities under a spatially explicit genotypic context^65^ for each species and sampling time (T1 and T2). Short analyses were carried out with K = 12 for all species, with 5,000,000 MCMC iterations, thinning each 1,000 iterations and burn-in of 10%, using the Akaike Information Criteria in MCMC context (AICM)^66^. Final simulations were carried out by fixing the number of most probable clusters (K) obtained in the first simulations for each species and sampling time, using 30,000,000 iterations of MCMC, thinning each 1,000 iterations and burn-in of 10%. In each analysis, we computed the Poisson-Voronoi tessellation maximizing the value for the rate of the Poisson process (λ)^67^. The number of genetic clusters was assessed through probability density distribution graphs, for each species in T1 and T2.

### Genetic comparison and population expansion

From the variations in the observed haplotype frequency, the genetic differentiation for each species and sampling time was compared applying an Analysis of Molecular Variance (AMOVA). The design of these analyses was carried out by grouping localities depending on the level of impact of the tsunami/earthquake (i.e., impacted area vs. northern and southern non-impacted areas). The genetic differentiation was assessed through AMOVAs based on matrices of estimated genetic distances according to the better-fitted sequence evolution model^68^ using the software Arlequin 3.5.2.2 ^69^. Evolution models of the sequences (estimated for each gene independently and for the concatenated data set), and the underlying parameters, were estimated with the program jModelTetst 2.1.10^70,71^. To investigate signals of population expansion in the three species at each sampling time (T1 and T2) we computed the distribution of pairwise differences from the segregating sites of the concatenated data set by calculating the mismatch distribution analyses implemented in DnaSP.

### Gene flow

It has been recently noted that Migrate-n can provide accurate results of recent migration rates, despite the fact that it has most frequently been used to explain long-term historical patterns^72^. Therefore, we used this software to calculate the genetic diversity and migration patterns of populations with the parameters effective population size (Θ) and migration rate among clusters (M) scaled by the mutation rate (μ) in Migrate-n 3.6.11^73–76^. The analyses were performed using 10 replicates of short chains with 50,000 iterations and one long chain with 1,000,000 iterations and 10% burn-in. The obtained values were used to parameterize the priors of Bayesian runs using six independent replicates for each species and sampling time. Each Bayesian run was performed with the transition/transversion ratio value of each gene obtained from the previously inferred evolution models. In each replicate we computed 5,000,000 iterations with samples taken every 1,000 steps and 10% burning. We compared the parameters obtained based on: (1) better adjustment considering constant or variable mutation rates for both genes, and (2) better adjustment to an exponential or uniform model of *a posteriori* distribution. For each type of mutation rate and distribution model, the six replicates were merged, and the associated marginal likelihood was compared under the Bayes Factor criterion for the selection of models^77^ implemented in Tracer 1.6.0 software^78^ with 10,000 Bootstrap replicates. The mean and standard deviation of the obtained Θ and M distributions were represented in gene flow diagrams using the Excel add-ins PopTools 3.2^79^. Additionally, the inferred population genetic structure was contrasted with a panmictic model, where it was assumed that all of the clusters form part of the same genetically interconnected unit. The adjustment was determined using Bayes Factor criterion estimated from the marginal likelihoods of both models using Tracer 1.6.0 software with 10,000 Bootstrap replicates.

## Supplementary information

Supplementary Information

## Data Availability

DNA sequences were uploaded to Genbank with Accession numbers MK914652 - MK917441.

## References

[1] Gardner TA, Cote IM, Gill JA, Grant A, Watkinson AR (2005). Hurricanes and Caribbean coral reefs: impacts, recovery patterns, and role in long-term decline. Ecology.

[2] Lugo AE (2008). Visible and invisible effects of hurricanes on forest ecosystems: an international review. Austral Ecology.

[3] Castilla JC, Manríquez PH, Camaño A (2010). Effects of rocky shore coseismic uplift and the 2010 Chilean mega earthquake on intertidal biomarker species. Mar. Ecol. Prog. Ser..

[4] Jaramillo E (2012). Ecological Implications of Extreme Events: Footprints of the 2010 Earthquake along the Chilean Coast. PLoS ONE.

[5] Castilla JC (1988). Earthquake-Caused Coastal Uplift and Its Effects on Rocky Intertidal Kelp Communities. Science.

[6] Putz FE, Sharitz RR (1991). Hurricane damage to old-growth forest in Congaree Swamp National Monument, South Carolina, USA. Can J For Res.

[7] Foster DR, Boose ER (1992). Patterns of forest damage resulting from catastrophic wind in central New England, USA. J Ecol.

[8] Merrens EJ, Peart DR (1992). Effects of hurricane damage on individual growth and stand structure in a hardwood forest in New Hampshire, USA. J Ecol.

[9] Allen BP, Pauley EF, Sharitz RR (1997). Hurricane impacts on liana populations in an old-growth southeastern bottomland forest. J Torrey Bot Soc.

[10] Platt WJ, Doren RF, Armentano TV (2000). Effects of hurricane Andrew on stands of slash pine (Pinus elliottii var. densa) in the everglades region of south Florida (USA). Plant Ecol.

[11] Boose ER, Chamberlin KE, Foster DR (2001). Landscape and regional impacts of hurricanes in New England. Ecol Monogr.

[12] Crabbe MJC (2008). Growth modelling indicates hurricanes and severe storms are linked to low coral recruitment in the Caribbean. Mar. Env. Res..

[13] Xi W, Peet RK, Urban DL (2008). Changes in forest structure, species diversity and spatial pattern following hurricane disturbance in a Piedmont North Carolina forest, USA. J Plant Ecol.

[14] Vargas G (2010). Coastal uplift and tsunami effects associated to the 2010 Mw8.8 Maule earthquake in Central Chile. Andean. Geology.

[15] Burger R, Lynch M (1995). Evolution and extinction in a changing environment: A quantitative-genetic analysis. Evolution.

[16] Lande R, Shannon S (1996). The role of genetic variation in adaptation and population persistence in a changing environment. Evolution.

[17] Hughes AR (2008). Ecological consequences of genetic diversity. Ecology letters.

[18] Lowe AJ, Boshier D, Ward M, Bacles CFE, Navarro C (2005). Genetic resource impacts of habitat loss and degradation; reconciling empirical evidence and predicted theory for neotropical trees. Heredity.

[19] Hall P, Walker S, Bawa K (1996). Effects of forest fragmentation on genetic diversity and mating system in a tropical tree, Pithecellobium elegans. Conserv Biol..

[20] Céspedes M, Gutierrez MV, Holbrook NM, Rocha OJ (2003). Restoration of genetic diversity in the dry forest tree Swietenia macrophylla (Meliaceae) after pasture abandonment in Costa Rica. Mol Ecol.

[21] Martínez EA, Cárdenas L, Pinto R (2003). Recovery and genetic diversity of the intertidal kelp Lessonia nigrescens (Phaeophyceae) 20 years after El Niño 1982/83. J. Phycol..

[22] Hirase S, Ikeda M, Hayasaka S, Iwasaki W, Kijima A (2016). Stability of genetic diversity in an intertidal goby population after exposure to tsunami disturbance. Mar Ecol..

[23] Miura O (2017). Ecological and genetic impact of the 2011 Tohoku Earthquake Tsunami on intertidal mud snails. Scientific reports.

[24] Ruegg JC (2009). Interseismic strain accumulation measured by GPS in the seismic gap between Constitución and Concepción in Chile. Physics of the Earth and Planetary Interiors.

[25] Fritz HM (2011). Field survey of the 27 February 2010 Chile Tsunami. Pure Appl Geophys.

[26] Chavanich S, Siripong A, Sojisuporn P, Menasveta P (2005). Impact of Tsunami on the seafloor and corals in Thailand. Coral Reefs.

[27] Kumaraguru AK, Jayakumar K, Jerald Wilson J, Ramakritinan CM (2005). Impact of the Tsunami of 26 December 2004 on the coral reef environment of Gulf of Mannar and Palk Bay in the southeast coast of India. Curr. Sci..

[28] Whanpetch N (2010). Temporal changes in benthic communities of seagrass beds impacted by a tsunami in the Andaman Sea, Thailand. Estuar. Coast. Shelf Sci..

[29] Lomovaskya BJ, Firstater FN, Gamarra Salazar A, Mendo J, Iribarne O (2011). Macro benthic community assemblage before and after the 2007 tsunami and earthquake at Paracas Bay, Peru. J. Sea Research.

[30] Mujica A (1993). Zooplancton en las aguas que circundan a Isla de Pascua. Ciencia y Tecnología del Mar..

[31] Thatje S, Fuentes V (2003). First record of anomuran and brachyuran larvae (Crustacea: Decapoda) from antartic waters. Polar Biology..

[32] Jaramillo E (1994). Patterns of species richness in sandy beaches of South America. South African Journal of Zoology..

[33] Contreras H, Duarte C, Jaramillo E, Fuentes N (2013). Morphometric variability in sandy beach crustaceans of Isla Grande de Chiloé, Southern Chile. Revista de Biología Marina y Oceanografía..

[34] Jaramillo E, Fuentealba S (1993). Down-shore zonation of two cirolanid isopods during two spirng-neap tidal cycles in a sandy beach of south central Chile. Revista Chilena de Historia Natural..

[35] Contreras H, Jaramillo E (2003). Geographical variation in natural history of the sandy beach isopod Excirolana hirsuticauda Menzies (Cirolanidae) on the Chilean coast. Estuarine, Coastal and Shelf Science..

[36] Jaramillo E, Carrasco F, Quijón P, Pino M, Contreras H (1998). Distribución y estructura comunitaria de la macroinfauna bentónica en la costa del norte de Chile. Revista Chilena de Historia Natural..

[37] Naylor E, Kennedy F (2003). Ontogeny of behavioural adaptations in beach crustaceans: some temporal considerations for integrated coastal zone. Estuarine, Coastal and Shelf Science..

[38] Baessolo L, Pérez-Schultheiss J, Aguilera A, Suazo C, Castro M (2010). Nuevos registros de Orchestoidea tuberculata Nicolet 1849 (Amphipoda, Talñitridae) en la costa de Chile. Hidrobiológica..

[39] Jaramillo E, Contreras H, Duarte C, Avellanal MH (2003). Locomotor activity and zonation of upper shore arthropods in a sandy beach of north central Chile. Estuarine, Coastal and Shelf Science..

[40] Scapini F (2014). Behaviour of mobile macrofauna is a key factor in beach ecology as response to rapid environmental changes. Estuarine, coastal and Shelf Science..

[41] Veas R (2013). The influence of environmental factors in the abundance and recruitment of the sand crab Emerita analoga (Stimp- son 1857): a source-sink dynamics?. Mar Environ Res.

[42] Sepúlveda RD, Valdivia N (2017). Macrobenthic Community Changes of Intertidal Sandy Shores after a Mega-Disturbance. Estuaries and coasts.

[43] Banks SC (2013). How does ecological disturbance influence genetic diversity? Trends in ecology &. evolution.

[44] Wright S (1931). Evolution in Mendelian populations. Genetics.

[45] Nei M (1975). The bottleneck effect and genetic variability in populations. Evolution.

[46] Allendorf FW (1986). Genetic drift and the loss of alleles versus heterozygosity. Zoo biology.

[47] Fraser DJ (2007). Comparative estimation of effective population sizes and temporal gene flow in two contrasting population systems. Molecular Ecology.

[48] Baldanzi S, McQuaid CD, Cannicci S, Porri F (2013). Environmental domains and range-limiting mechanisms: testing the Abundant Centre Hypothesis using Southern African sandhoppers. PLoS One.

[49] Ketmaier V, Matthaeis ED, Fanini L, Rossano C, Scapini F (2010). Variation of genetic and behavioural traits in the sandhopper Talitrus saltator (Crustacea Amphipoda) along a dynamic sand beach. Ethology Ecology &. Evolution.

[50] Fanini L (2017). Life-history, substrate choice and Cytochrome Oxidase I variations in sandy beach peracaridans along the Rio de la Plata estuary. Estuarine, Coastal and Shelf Science.

[51] Sepúlveda RD, Valdivia N (2016). Localised effects of a mega-disturbance: spatiotemporal responses of intertidal sandy shore communities to the 2010 Chilean earthquake. PloS one.

[52] Wang IJ (2010). Recognizing the temporal distinctions between landscape genetics and phylogeography. Molecular Ecology.

[53] Wang IJ (2011). Choosing appropriate genetic markers and analytical methods for testing landscape genetic hypotheses. Molecular Ecology.

[54] Bohonak AJ, Vandergast AG (2011). The value of DNA sequence data for studying landscape genetics. Molecular Ecology.

[55] Sambrook, J., Fritschi, E. F. & Maniatis, T. *Molecular cloning: a laboratory manual* (Cold Spring Harbor Laboratory Press, 1989).

[56] Pavesi L (2013). Genetic connectivity between land and sea: the case of the beachflea Orchestia montagui (Crustacea, Amphipoda, Talitridae) in the Mediterranean Sea. Frontiers in Zoology.

[57] Yang L, Hou Z, Li S (2013). Marine incursion into East Asia: a forgotten driving force of biodiversity. Proc R Soc B.

[58] Giribet, G. *et al*. The position of crustaceans within the Arthropoda - evidence from nine molecular loci and morphology. Crustacean Issues 16: Crustacea and Arthropod Relationships. Festschrift for Frederick R. Schram. S. Koenemann and R. A. Jenner. Boca Raton (Taylor & Francis, 2005).

[59] Maddison, W. P. & Maddison, D. R. Mesquite: a modular system for evolutionary analysis. Versión 3.4. http://mesquiteproject.org (2018).

[60] Xia X, Xie Z, Salemi M, Chen L, Wang Y (2003). An index of substitution saturation and its application. Molecular Phylogenetics and Evolution..

[61] Tajima F (1989). Statistical method for testing the neutral mutation hypothesis by DNA polymorphism. Genetics..

[62] Xia X (2017). DAMBE6: New tools fpr microbial genomics, phylogenetics and molecular evolution. Journal of Heredity..

[63] Rozas J (2017). DnaSP v6: DNA sequence polymorphism analysis of large datasets. Molecular Biology ans Evolution..

[64] StatSoft. Inc. STATISTICA (data analysis software system). Version 7, www.statsoft.com. (2004).

[65] Guillot G, Estoup A, Mortier F, Cosson JF (2005). A spatial model for landscape genetics. Genetics..

[66] Baele G, Sibon WL, Drummond AJ, Suchard MA, Lerney P (2012). Accurate model selection of relaxed molecular clocks in Bayesian phylogenetics. Molecular Biology and Evolution..

[67] Lantuéjoul, C. Geostatistical simulations. Models and algorithms. Springer, Berlin. 256 pp (2013).

[68] Excoffier L, Smouse PE, Quattro JM (1992). Analysis of molecular variance inferred from metric distances among dna haplotypes: Application to human mitochondrial DNA restriction data. Genetics..

[69] Excoffier L, Lischer HEL (2010). Arlequin suite ver 3.5: A new series of programs to perform population genetics analyses under Linux and Windows. Molecular Ecology Resources..

[70] Guindon S, Gascuel O (2003). A simple, fast and accurate methods to estimate large phylogenies by maximum-likelihood. Systematic Biology..

[71] Darriba D, Taboada GL, Doallo R, Posada D (2012). jModelTest 2: more models, new heuristics and parallel computing. Nature Methods..

[72] Samarasin P (2017). The problem of estimating recent genetic connectivity in a changing world. Conservation Biology.

[73] Kuhner MK, Yamato J, Felsenstein J (1995). Estimating effective population size and mutation rate from sequence data using Metropolis-Hastings sampling. Genetics.

[74] Beerli P, Felsenstein J (1999). Maximum-likelihood estimation of migration rates and effective population numbers in two populations using a coalescent approach. Genetics..

[75] Kingman JFC (2000). Origins of the Coalescent: 1974–1982. Genetics.

[76] Beerli, P. How to use migrate or why are Markov Chain Monte Carlo programs difficult to use? In: Population Genetics for Animal Conservation, volume 17 of Conservation Biology. (Bertorelle, G; M.W. Bruford, H.C. Hauffe, A. Rizzoli & C. Vernesi, eds) (Cambridge University Press, 2009).

[77] Gelman, A., Carlin, J. B., Stern, H. S. & Rubin, D. B. *Bayesian data analysis* (Chapman & Hall, London, 1995).

[78] Rambaut, A. & Drummond, A. J. Tracer version 1.6. Available in, http://beast.bio.ed.ac.uk/Tracer (2007).

[79] Hood, G. M. PopTools version 3.2.5, http://www.poptools.org (2010).

