# Supplementary material for: Post-Disturbance Genetic Changes: The Impact of the 2010 Mega-Earthquake and Tsunami on Chilean Sandy Beach Fauna
Source: Sci Rep. 2019 Oct 2;9:14239. doi: 10.1038/s41598-019-50525-1 (PMC6775079; doi:10.1038/s41598-019-50525-1)
Supplement: Supplementary file 1 — Supplementary Information [file 41598_2019_50525_MOESM1_ESM.docx]

**POST-DISTURBANCE GENETIC CHANGES: THE IMPACT OF THE 2010 MEGA-EARTHQUAKE AND TSUNAMI ON CHILEAN SANDY BEACH FAUNA**

Antonio Brante, Garen Guzmán-Rendón, Erwin M. Barría, Marie Laure Guillemin, Iván Vera-Escalona & Cristián E. Hernández

Table S1. Molecular Analysis of Variance (AMOVA) using the T1 and T2 data sets for three common crustacean species of Chilean sandy beach ecosystems: *Emerita analoga*, *Excirolana hirsuticauda*, and *Orchestoidea tuberculata*. The populations were grouped depending on the level of impact of the tsunami/earthquake (i.e., impacted area, northern non-impacted areas, and southern non-impacted areas); T1 = sampling performed one month after the 27F earthquake and T2 = sampling performed three years after the 27F earthquake

|  | |  |  | T1 | | | |  | T2 | | | |
| --- | --- | --- | --- | --- | --- | --- | --- | --- | --- | --- | --- | --- |
|  | Species | Source of variation (*) |  | Component of variance | % Total | p-value | φ |  | Component of variance | % Total | p-value | Φ |
| *Emerita analoga* | | Va |  | 0.086 | 4.83 | **<0.001** | φ_CT_: 0.048 |  | 0.094 | 4.33 | **<0.001** | Φ_CT_: 0.043 |
|  | | Vb |  | 0.210 | 11.76 | **<0.001** | φ_SC_: 0.124 |  | 0.265 | 12.16 | **<0.001** | Φ_SC_: 0.127 |
|  | | Vc |  | 1.487 | 83.41 | **<0.001** | φ_ST_: 0.165 |  | 1.817 | 83.51 | **0.035** | Φ_ST_: 0.165 |
|  | |  |  |  | | | |  |  | | | |
| *Excirolana hirsuticau*da | | Va |  | 0.140 | 5.47 | **<0.001** | φ_CT_: 0.055 |  | 0.157 | 6.14 | **<0.001** | Φ_CT_: 0.061 |
|  | | Vb |  | 0.214 | 8.31 | **<0.001** | φ_SC_: 0.088 |  | 0.220 | 8.58 | **<0.001** | Φ_SC_: 0.091 |
|  | | Vc |  | 2.220 | 86.22 | **0.044** | Φ_ST_: 0.138 |  | 2.180 | 85.27 | **0.041** | Φ_ST_: 0.147 |
|  | |  |  |  | | | |  |  | | | |
| *Orchestoidea tuberculata* | | Va |  | 0.111 | 6.09 | **<0.001** | Φ_CT_: 0.061 |  | 0.183 | 9.65 | **<0.001** | Φ_CT_: 0.096 |
|  | | Vb |  | 1.229 | 66.85 | **<0.001** | Φ_SC_: 0.712 |  | 0.976 | 51.54 | **<0.001** | Φ_SC_: 0.570 |
|  | | Vc |  | 0.497 | 27.09 | 0.217 | Φ_ST_: 0.729 |  | 0.735 | 38.81 | **0.046** | Φ_ST_: 0.611 |

(*) **Va**: Variation among zones; **Vb**: Variation between localities within zones; **Vc**: Variation within localities. Bold p-value = significant values.

Table S2. Genetic diversity estimates for three common crustacean species of Chilean sandy beach ecosystems: *Emerita analoga*, *Excirolana hirsuticauda*, and *Orchestoidea tuberculata*. The localities in bold correspond to the impacted area and the localities that are above and below these correspond to the northern non-impacted area and the southern non-impacted area, respectively. Two sample times: T1 = sampling performed one month after the 27F earthquake and T2 = sampling performed three years after the 27F earthquake.


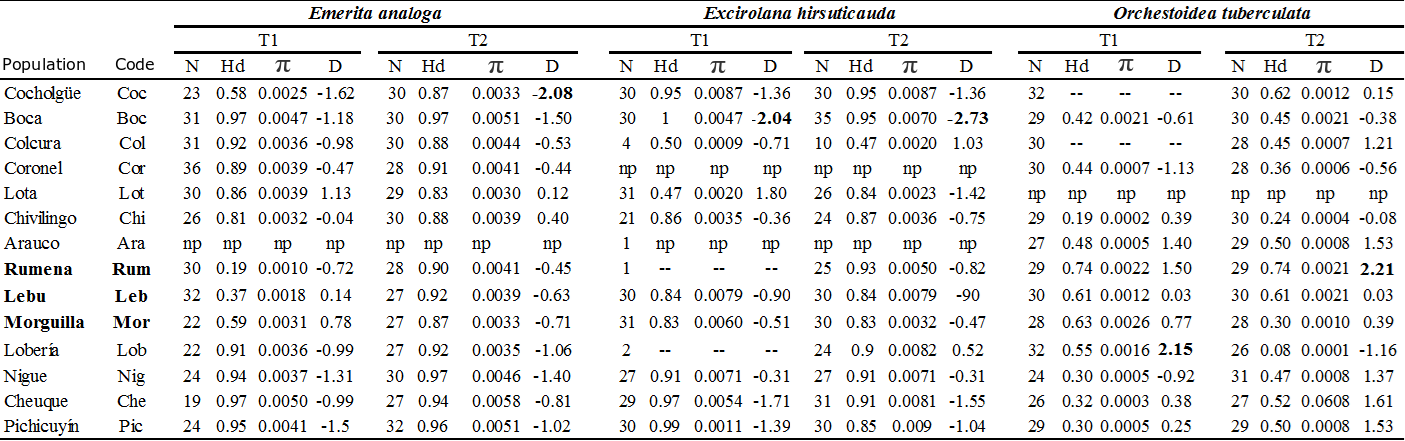


N: number of concatenated sequences (i.e., including the mitochondrial gene COI and the nuclear gene 16S) used in the analyses; Hd: haplotype diversity; π: nucleotide diversity; D: Tajima's D, bold values show significant differences. np = not present
